# Diagnostic significance of serum *PP4R1* and its predictive value for the development of chronic complications in patients with type 2 diabetes mellitus

**DOI:** 10.1186/s13098-021-00642-7

**Published:** 2021-03-09

**Authors:** Wenjing Li, Lanbo Peng, Chao Yang, Guangmin Chen

**Affiliations:** 1 Endocrinology Department, The Second People’s Hospital of Liaocheng, Liaocheng, Shandong 252600 China; 2 Endocrinology Department, Liaocheng People’s Hospital, Liaocheng, Shandong 252000 China; 3 Nephrology and Urology Centre, University-Town Hospital of Chongqing Medical University, No. 55, University-Town Middle Road, Chongqing, 401551 China

**Keywords:** *PP4R1*, T2DM, Chronic complications, Diagnosis

## Abstract

**Background:**

Protein phosphatase 4 regulatory subunit 1 (PP4R1) is one of the regulatory subunits of PP4. It has been determined to be involved in the regulation of TNF-α-induced hepatic insulin resistance and gluconeogenesis. Considering the important role of PP4R1 in hepatic insulin resistance, the current study explored the expression and diagnostic value of *PP4R1* in type 2 diabetes mellitus (T2DM) patients and further investigated its predictive value for the development of chronic complications.

**Method:**

Hundred and five patients with T2DM and 97 healthy controls were collected. qRT-PCR was used for the measurement of serum *PP4R1* mRNA level in both T2DM and control groups. The diagnostic value of PP4R1 in T2DM patients was evaluated using receiver operating characteristic (ROC) curve. Kaplan-Meier methods and Cox regression analysis were used to evaluate the predictive value of *PP4R1* for the development of chronic complications in T2DM patients.

**Results:**

*PP4R1* was determined to be elevated in the serum of T2DM patients compared with healthy controls. Serum *PP4R1* had the potential to distinguish T2DM patients from healthy controls with a sensitivity of 81.9% and specificity of 82.5%. Patients with high *PP4R1* expression experienced more chronic complications events. The multivariate Cox analysis results suggested that serum *PP4R1* expression was an independent factor for the occurrence of chronic complications for T2DM patients.

**Conclusion:**

*PP4R1* is elevated in the serum of T2DM patients, had the potential to distinguish T2DM patients from healthy controls. *PP4R1* serves as a promising biomarker for predicting the risk of future chronic complications in T2DM patients.

## Introduction

Type 2 diabetes mellitus (T2DM) is a metabolic disorder, characterized by hyperglycemia in the context of reduced insulin sensitivity and insulin resistance. Approximately 90 % of diabetes cases are diagnosed to be T2DM [1]. In recent years, the global diabetes epidemic continues to increase, and T2DM has been reported to reach epidemic proportions [2, 3]. In China, approximately 100 million individuals suffer from T2DM, accounting for about a quarter of the global T2DM population [4]. And the number of T2DM cases in China will reach about 438 million in 2030 [4]. In addition to a rapid increase in prevalent cases, the management weaknesses for diabetes attract our attention in China [5]. And a growing number of cases with T2DM will develop into diabetic complications. As a result of the rising prevalence of chronic complications of T2DM, the challenge of diabetes care has increased [6]. At present, the oral glucose tolerance test (OGTT) is the gold standard for the diagnosis of diabetes. Some cases may not adopt the detection because of the complicate detection process, which may lead to a certain degree of misdiagnosis. The development of biomarkers for early prediction of T2DM will be beneficial to delay the occurrence or control the severity of the diseases.

T2DM affects the homeostasis of glucose metabolism, and hyperglycemia is characteristic of T2DM [7, 8]. To date, the causes of T2DM are still not fully understood, and a definitive cure remains unavailable [9, 10]. It is well known that hepatic insulin resistance is a predominant cause of T2DM. Protein phosphatase 4 regulatory subunit 1 (PP4R1) is one of the regulatory subunits of PP4, involving in critical cellular processes, such as microtubule growth and organization, DNA damage checkpoint recovery, apoptosis, and tumor necrosis factor-alpha signaling [11]. PP4R1 has been determined to be involved in the regulation of TNF-α-induced hepatic insulin resistance [12]. Consistently, another study further confirmed that inhibition of PP4R1 rescued the effect of TNF-α on the generation of glycogen and regulated the activation of the insulin signaling pathway, suggesting the crucial role of PP4R1 in T2DM [13]. Additionally, Wang et al. also reported that downregulation of PP4R1 significantly attenuated the increased level of glucose production in murine liver cells after treatment with TNF-α, indicating that PP4R1 participants in the regulation of TNF-α-induced gluconeogenesis [14]. Considering the important role of hepatic insulin resistance in the occurrence of T2DM, we concluded that PP4R1 may be associated with the onset or development of T2DM. However, the expression profile of PP4R1 in T2DM has not been determined.

Therefore, the current study was performed to explore the expression and diagnostic value of PP4R1 in T2DM patients, and further investigate its predictive value for the development of chronic complications.

## Materials and methods

### Study population and sample collection

Hundred and five patients with T2DM were recruited from The Second People’s Hospital of Liaocheng, all patients were newly diagnosed with T2DM according to the American Diabetic Association (ADA) guidelines [15]: a fasting blood glucose level ≥ 7 mmol/l, a 2-h postprandial blood glucose level ≥ 11.1 mmol/l or a glycated hemoglobin level ≥ 6.5 %. 97 healthy controls were collected as the control group, and the inclusion criteria were as follows: (1) a fasting blood glucose level < 7 mmol/l and a glycated hemoglobin level < 6.0 %; (2) no history of diabetes or other autoimmune diseases in the degree relatives; (3) no hypertension. None of the subjects were receiving any antidiabetic, antihypertensive, or hypolipidemic drugs. 5 ml peripheral venous blood was collected from each participant and was allowed to coagulate for 30 min, then centrifuged at 800 ×*g* for 7 min, finally the serum was stored at − 80 ℃ for subsequent experiments.

This study design was approved and supported by the Ethical Committee of The Second People’s Hospital of Liaocheng, and each participant signed written informed consent.

### Follow‐up survey

The T2DM patients were followed up for 5 years, and the progression of chronic complications was recorded, including microvascular complications and macrovascular complications. For microvascular complications, patients with microalbuminuria ≥ 20 mg/dl were diagnosed to suffer from microvascular complications. Fundus was examined using an ophthalmoscope by an ophthalmologist. For macrovascular complications, patients with lower extremity or carotid intimal medial thickness ≥ 1 mm or atherosclerosis and arteriosclerosis were diagnosed to suffer from macrovascular complications.

### Quantitative real time-polymerase chain reaction (qRT-PCR) assay

Total RNA was extracted using Trizol reagent (Invitrogen, Carlsbad, CA, USA). A High Capacity cDNA Reverse Transcription Kit (Applied Biosystems, Foster City, CA, USA) was used for reverse transcription. The SYBR green I Master Mix kit (Invitrogen, Carlsbad, CA, USA) was applied for the detection of the *PP4R1* mRNA level, which was normalized to GAPDH. The following thermocycling conditions were used for the PCR: Initial denaturation at 95 ˚C for 60 s, 40 cycles of 95 ˚C for 5 s, 60 ˚C for 20 s. The relative *PP4R1* mRNA expression level was determined using the comparative delta CT (2^−ΔΔCt^) method. Primers were as follows: PP4R1, 5′-ACGTCCCATTGCTCTGAATC-3′ (forward), 5′-CTTGGGACATCTGCCAAAGT-3′ (reverse); GAPDH, 5′-TGCACCACCAACTGCTTAGC-3′ (forward), 5′-GGCATGGACTGTGGTCATGAG-3′ (reverse).

### Statistical analysis

SPSS version 18.0 software (SPSS Inc., Chicago, IL, USA) and GraphPad Prism 5.0 software (GraphPad Software, Inc., USA) were applied for data analysis. Differences between two groups were compared using the χ^2^ test, or *t*-test. Kaplan-Meier methods and Cox regression analysis were used to evaluate the predictive value of *PP4R1* for the development of chronic complications in T2DM patients. The receiver operating characteristic (ROC) curve was established, and the area under curve (AUC) with 95 % confidence interval (CI) was detected to assess the discrimination ability of *PP4R1* between T2DM patients and healthy controls. *P* < 0.05 was identified to be statistically significant.

## Results

### Basic features

The clinical information of the study population was recorded in Table 1. There was no significant difference in the distribution of age (*P* = 0.536), gender (*P* = 0.997) and BMI (*P* = 0.096) between T2DM patients’ group and healthy controls. The values of fasting blood glucose, TC, TG, and LDL were higher in the T2DM patients’ group than that in control group, while HDL was lower in the T2DM patients’ group, all differences reached significant levels (all *P* < 0.05).

**Table 1:** Characteristics of the study population

Variables	Control (n = 97)	T2DM (n = 105)	*P* value
Age	51.06 ± 4.71	51.50 ± 5.19	0.536
Gender (female/male)	36/61	39/66	0.997
BMI (kg/m^2^)	24.73 ± 2.44	25.30 ± 2.40	0.096
Fasting blood glucose	5.08 ± 0.65	7.74 ± 0.43	< 0.001
TC (mmol/l)	4.56 ± 1.06	4.89 ± 1.00	0.026
TG (mmol/l)	1.92 ± 0.41	2.11 ± 0.35	< 0.001
HDL (mmol/l)	1.35 ± 0.12	1.29 ± 0.12	< 0.001
LDL (mmol/l)	2.37 ± 0.35	2.65 ± 0.36	< 0.001

*BMI* body mass index, *TC* total cholesterol, *TG* triglyceride, *HDL* high-density lipoprotein, *LDL* low-density lipoprotein

### Serum level of *PP4R1* in T2DM patients

**Fig. 1:**
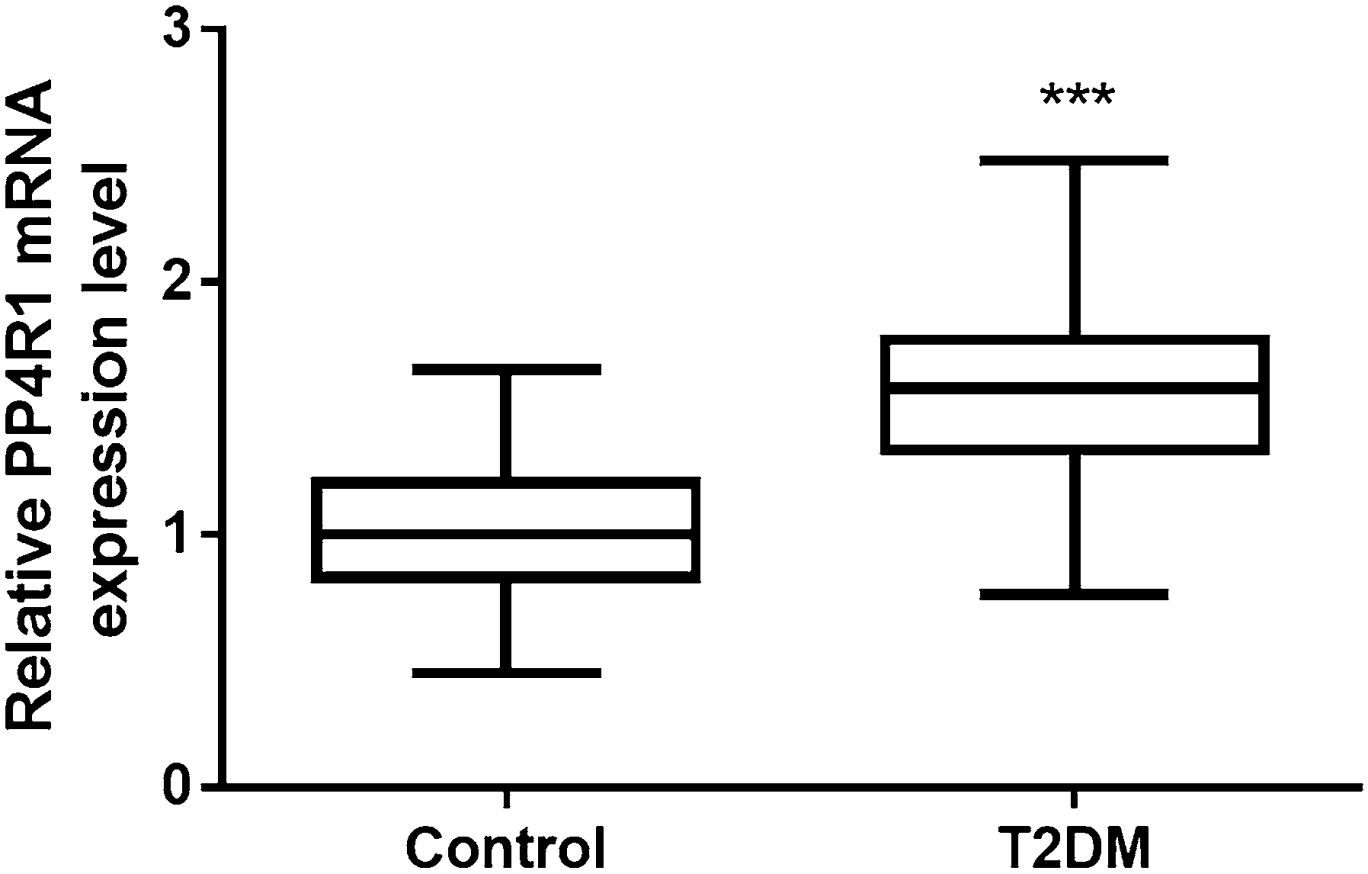
The level of *PP4R1* increased significantly in the serum of T2DM patients compared with the control group. *** *P* < 0.001

A total of 97 healthy controls and 105 T2DM patients were included in the current study. The serum expression level of *PP4R1* was measured by using qRT-PCR in the study population and compared by using an independent Student’s t test. As shown in Fig. 1, the level of *PP4R1* increased significantly in the T2DM patients’ group compared with the control group (*P* < 0.001).

### Diagnostic value of *PP4R1* expression for T2DM patients

**Fig. 2:**
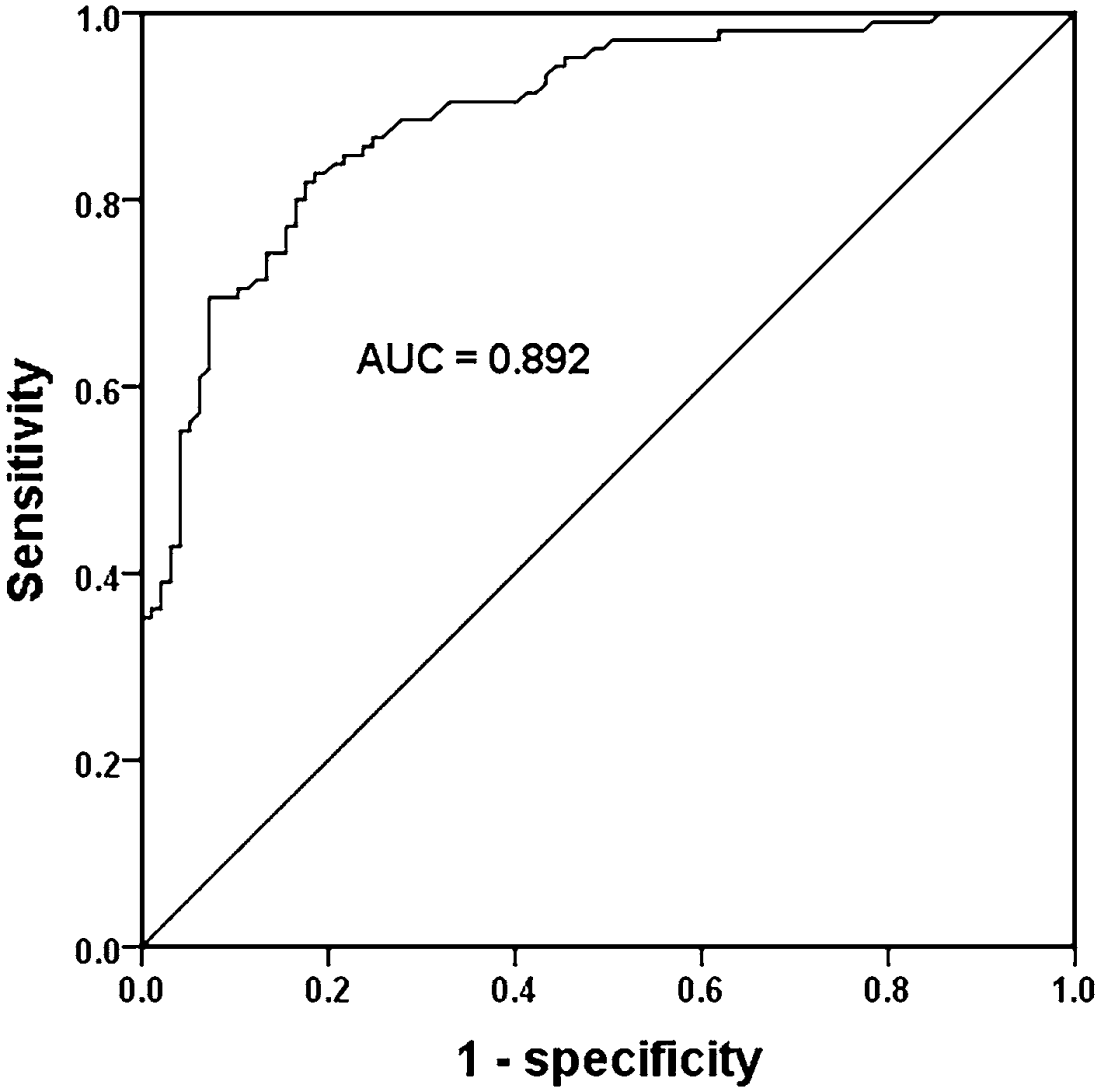
Diagnostic value of serum *PP4R1* in T2DM patients. The AUC was 0.892, with a sensitivity of 81.9 % and specificity of 82.5 % at the cutoff value of 1.2645

According to the levels of PP4R1 in the serum of T2DM and control groups, the ROC curve was constructed to assess the diagnostic value of serum *PP4R1* in T2DM patients. As shown in Fig. 2, the AUC was 0.892, with a sensitivity of 81.9% and specificity of 82.5% at the cutoff value of 1.2645. The results suggested that serum *PP4R1* was a valuable biomarker for differentiating T2DM patients from healthy controls.

### Prognostic value of ***PP4R1*** for chronic complications in T2DM patients

**Fig. 3:**
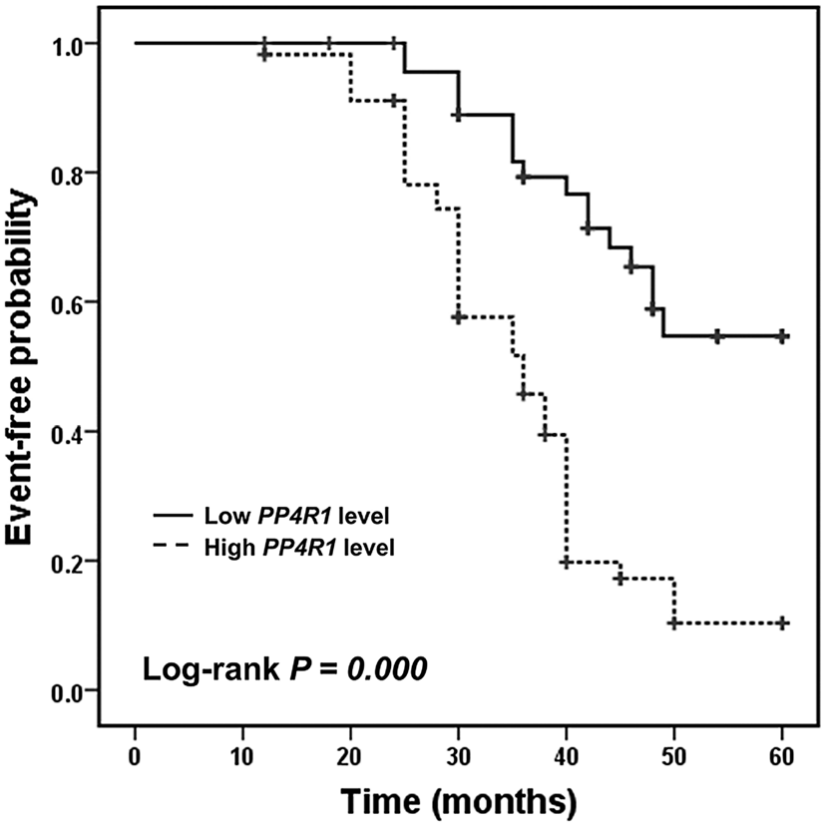
The Kaplan-Meier analysis was performed to evaluate the predictive value of serum *PP4R1* for the occurrence of chronic complications in T2DM patients. Patients with high *PP4R1* expression experienced more chronic complications events (log-rank *P* = 0.000)

To investigate the predictive value of *PP4R1* for the development of chronic complications in patients with T2DM, the Kaplan-Meier survival curve was established. The 105 T2DM patients were followed up for five years, and neither death nor acute complications occurred. During the follow-up, a total of 61 patients were observed to suffer from chronic complications. In all, 44 patients had macrovascular complications, 26 patients had microalbuminuria, among them 7 patients were diagnosed with diabetic retinopathy. The Kaplan-Meier survival results indicated that patients with high *PP4R1* expression experienced more chronic complications events (log-rank *P* = 0.000, Fig. 3). Additionally, we further detected the influence of clinical parameters on the occurrence of chronic complications. The multivariate Cox analysis results suggested that serum *PP4R1* expression was an independent factor for the occurrence of chronic complications for T2DM patients (HR = 3.527, 95 % CI = 1.977–6.291, *P* = 0.000; Table 2).

**Table 2:** Multivariate Cox regression analysis for serum *PP4R1* in T2DM patients

Variables	Multivariate analysis		
	HR	95 % CI	*P* value
*PP4R1*	3.527	1.977–6.291	0.000**
Age	1.015	0.559–1.843	0.960
Gender	1.254	0.710–2.215	0.435
BMI	1.075	0.619–1.868	0.797
Fasting blood glucose	1.413	0.804–2.483	0.229
TC	1.663	0.872–3.173	0.123
TG	1.745	0.914–3.330	0.091
HDL	1.712	0.965–3.037	0.066
LDL	1.460	0.795–2.684	0.223

*BMI* body mass index, *TC* total cholesterol, *TG* triglyceride, *HDL* high-density lipoprotein, *LDL* low-density lipoprotein

**P < 0.01.

## Discussion

T2DM is becoming a great public health problem, which is characterized by elevated blood glucose and attenuated insulin action [16, 17]. T2DM affects the homeostasis of glucose metabolism and further results in serious complications, such as diabetic nephropathy and diabetic nephropathy [18]. With the prevalence of T2DM increased year by year, the morbidity and mortality of cardiovascular disease related to diabetes continue to rise, which have a great influence on important social, financial and health system implications [17]. Abnormal metabolism of glucose and lipoid is one of the main physiological characteristics of T2DM, leading to hyperlipidemia and various microvascular and macrovascular T2DM complications over the long term [19]. In the present study, 97 healthy controls and 105 patients who were newly diagnosed with T2DM were recruited. Analysis of their clinical information revealed the changes in serum lipid levels between T2DM patients and healthy controls, reflecting the development tendency of chronic complications in T2DM patients. Without early interventions and adequate management, those patients will develop diabetic complications in a higher proportion [20]. As a recent study in China reported, neuropathy, retinopathy, and nephropathy were detected in 29%, 25%, and 16% of all T2DM patients, respectively [21, 22]. At present, OGTT is the gold standard for the diagnosis of diabetes. As a result of the complicated detection process, some cases do not coordinate the detection, leading to a certain of misdiagnosis [23]. It is quite necessary to explore simple and efficient diagnosis methods.

Recently, non-traditional marker attracts researchers’ attention to be adjuncts of standard measures of T2DM patients [24]. The serum-based circulating biomarker is a novel and important diagnostic tool. As a result of non-invasive, circulating biomarkers attract great concern in recent years. For example, an elevated level of serum proteinase 3 (PR3) was determined in latent autoimmune diabetes mellitus in adults (LADA) patients by Yu et al. suggesting PR3 to be a potential biomarker for differentiating LADA and T2DM patients [25]. As Acharya et al. reported that serum Soluble Tumor Necrosis Factor-like Weak inducer of apoptosis (sTWEAK) level was lower in chronic periodontitis (CP) with T2DM patients than both CP and healthy individuals, and showed significant association with glycemic levels, indicating its potential role in T2DM [26]. Another study by Huang et al. reported that serum haptoglobin (Hp) levels were significantly higher in subjects with diabetic kidney disease (DKD) than controls, and associated with serum creatinine levels, it is suggested that serum Hp levels may be used as a potential biomarker for the early diagnosis and monitoring of DKD in T2DM patients [27]. In the current study, PP4R1 was identified to be highly expressed in the serum of T2DM patients, suggesting its potential role in the disease progression. Furthermore, the ROC curve results determined *PP4R1* to be a valuable biomarker for differentiating T2DM patients from healthy controls. However, in the present study, only the mRNA levels of PP4R1 were detected in the serum of T2DM patients, in future studies, the protein levels of PP4R1 should be further confirmed.

PP4R1 is one of the regulatory subunits of PP4, it is considered to be a regulator of TNF-α-induced hepatic insulin resistance [12]. In the liver, glucose metabolism homeostasis contains two major processes, including gluconeogenesis and glycogenesis [28, 29]. The impairment of insulin sensitivity in the liver can result in increased gluconeogenesis and decreased glycogenesis [30]. As previous evidence described, PP4R1 is involved in the regulation of TNF-α-induced gluconeogenesis, and PP4R1 downregulation inhibits glucose production in murine liver cells after treatment with TNF-α [14]. In the present study, PP4R1 was determined to be elevated in the serum of T2DM patients and had the potential to distinguish T2DM patients from healthy controls, which was supported by the previous evidence. T2DM affects the homeostasis of glucose metabolism and further results in serious complications, such as diabetic nephropathy and diabetic nephropathy. Based on the fact of the crucial role of PP4R1 in T2DM patients, we further investigated its predictive value for the development of chronic complications in patients with T2DM. In the current study, 105 T2DM patients were followed up for five years. And during the follow-up, neither death nor acute complications occurred, a total of 61 patients were observed to suffer from chronic complications, including 44 macrovascular complications, 26 microalbuminuria, and 7 diabetic retinopathy. Furthermore, the Kaplan-Meier survival results indicated that patients with high *PP4R1* expression experienced more chronic complications events, and serum *PP4R1* expression was an independent factor for the occurrence of chronic complications for T2DM patients. These data provide evidence for *PP4R1* as a promising biomarker for predicting the risk of future chronic complications in T2DM patients.

It is known that PP4R1 is one of the regulatory subunits of PP4, which has been considered as a critical regulator in TNF-α induced hepatic insulin resistance [12]. As Lin et al. reported, PP4R1 is involved in miR-338-3p mediated insulin resistance [13]. In addition, it is reported that knockdown of PP4R1 is suggested to alleviate TNF-α induced generation of glycogen and inhibit the activation of the AKT-GSK3β pathway [13]. In consideration of the previous evidence and the present results, we speculated that PP4R1 might contribute to the occurrence of T2DM through regulating the AKT-GSK3β pathway. However, future studies are needed to verify our hypothesis.

## Conclusions

Taken together, PP4R1 was determined to be elevated in the serum of T2DM patients, had the potential to distinguish T2DM patients from healthy controls. *PP4R1* serves as a promising biomarker for predicting the risk of future chronic complications in T2DM patients.

## Data Availability

The datasets used and/or analysed during the current study are available from the corresponding author on reasonable request.

## References

[1] Zheng Y, Ley SH, Hu FB (2018). Global aetiology and epidemiology of type 2 diabetes mellitus and its complications. Nat Rev Endocrinol.

[2] Liu Y, Zhang D, Yuan J, Song L, Zhang C, Lin Q (2020). Hyperbaric oxygen ameliorates insulin sensitivity by increasing GLUT4 expression in skeletal muscle and stimulating UCP1 in brown adipose tissue in T2DM mice. Front Endocrinol (Lausanne).

[3] Bhatia P, Raina S, Chugh J, Sharma S (2015). miRNAs: early prognostic biomarkers for Type 2 diabetes mellitus?. Biomark Med.

[4] Liu D, Pan JM, Pei X, Li JS (2020). Interaction between apolipoprotein M gene single-nucleotide polymorphisms and obesity and its effect on Type 2 diabetes mellitus susceptibility. Sci Rep.

[5] Pang X, Yin P, Han J, Wang Z, Zheng F, Chen X (2019). cPLA2a correlates with metastasis and poor prognosis of osteosarcoma by facilitating epithelial-mesenchymal transition. Pathol Res Pract.

[6] Jiang YY, Liu M, Ji N, Zeng XY, Dong WL, Mao F (2019). Disease burden of diabetes attributable to high body mass index in China,1990–2016. Zhonghua Liu Xing Bing Xue Za Zhi.

[7] Seboko AM, Conradie MM, Kruger MJ, Ferris WF, Conradie M, van de Vyver M (2018). Systemic factors during metabolic disease progression contribute to the functional decline of adipose tissue-derived mesenchymal stem cells in reproductive aged females. Front Physiol.

[8] DeFronzo RA, Ferrannini E, Groop L, Henry RR, Herman WH, Holst JJ (2015). Type 2 diabetes mellitus. Nat Rev Dis Primers.

[9] Hong S, Chang Y, Jung HS, Yun KE, Shin H, Ryu S (2017). Relative muscle mass and the risk of incident type 2 diabetes: a cohort study. PLoS One.

[10] American Diabetes A (2010). Diagnosis and classification of diabetes mellitus. Diabetes Care.

[11] PP4R1 (2020). Accelerates cell growth and proliferation in HepG2 hepatocellular carcinoma (expression of concern). Onco Targets Ther.

[12] Zhao H, Huang X, Jiao J, Zhang H, Liu J, Qin W (2015). Protein phosphatase 4 (PP4) functions as a critical regulator in tumor necrosis factor (TNF)-alpha-induced hepatic insulin resistance. Sci Rep.

[13] Dou L, Wang S, Sun L, Huang X, Zhang Y, Shen T (2017). Mir-338-3p mediates Tnf-A-induced hepatic insulin resistance by targeting PP4r1 to regulate PP4 expression. Cell Physiol Biochem.

[14] Wang S, Li L, Chen X, Huang X, Liu J, Sun X (2018). miR3383p mediates gluconeogenesis via targeting of PP4R1 in hepatocytes. Mol Med Rep.

[15] American Diabetes A (2014). Standards of medical care in diabetes-2014. Diabetes Care.

[16] Shi L, Feng L, Yang Y, Li X, Zhang M, Zhang Y (2018). Prevention of type 2 diabetes mellitus with acupuncture: protocol for a systematic review and meta-analysis. Medicine.

[17] Ogurtsova K, da Rocha Fernandes JD, Huang Y, Linnenkamp U, Guariguata L, Cho NH (2017). IDF diabetes atlas: global estimates for the prevalence of diabetes for 2015 and 2040. Diabetes Res Clin Pract.

[18] Wu H, Eggleston KN, Zhong J, Hu R, Wang C, Xie K (2018). How do type 2 diabetes mellitus (T2DM)-related complications and socioeconomic factors impact direct medical costs? A cross-sectional study in rural Southeast China. BMJ Open.

[19] Zhou P, Xie W, He S, Sun Y, Meng X, Sun G, et al. Ginsenoside Rb1 as an Anti-Diabetic Agent and Its Underlying Mechanism Analysis. Cells. 2019;8(3). 10.3390/cells8030204 10.3390/cells8030204 PMC6468558 30823412

[20] Li MZ, Su L, Liang BY, Tan JJ, Chen Q, Long JX (2013). Trends in prevalence, awareness, treatment, and control of diabetes mellitus in mainland china from 1979 to 2012. Int J Endocrinol.

[21] Zhao H, Shu L, Huang W, Wang W, Song G (2019). Difference analysis of related factors in macrovascular and microvascular complications in Chinese patients with Type 2 diabetes mellitus: a case-control study protocol. Diabetes Metab Syndr Obes.

[22] Pan C, Yang W, Jia W, Weng J, Tian H (2009). Management of Chinese patients with type 2 diabetes, 1998–2006: the Diabcare-China surveys. Curr Med Res Opin.

[23] Meijnikman AS, De Block CEM, Dirinck E, Verrijken A, Mertens I, Corthouts B (2017). Not performing an OGTT results in significant underdiagnosis of (pre)diabetes in a high risk adult Caucasian population. Int J Obes (Lond).

[24] Osei K, Gaillard T (2017). Disparities in cardiovascular disease and Type 2 diabetes risk factors in blacks and whites: dissecting racial paradox of metabolic syndrome. Front Endocrinol (Lausanne).

[25] Yu Y, Liu LL, Xiao XY, Wang YD, Xu AM, Tu YT (2019). Changes and clinical significance of serum proteinase 3 in latent autoimmune diabetes in adults. Zhonghua Yi Xue Za Zhi.

[26] Acharya AB, Chandrashekar A, Acharya S, Shettar L, Thakur S (2019). Serum sTWEAK levels in chronic periodontitis and type 2 diabetes mellitus. Diabetes Metab Syndr.

[27] Huang Y, Huang Y, Zhang R, Jin L, Zhang H, Hu C (2019). Serum haptoglobin levels are associated with renal function decline in type 2 diabetes mellitus patients in a Chinese Han population. Diabetes Res Clin Pract.

[28] Shao Y, Fu YX, Wang QF, Cheng ZQ, Zhang GY, Hu SY (2019). Khubchandani’s procedure combined with stapled posterior rectal wall resection for rectocele. World J Gastroenterol.

[29] Wahren J, Ekberg K (2007). Splanchnic regulation of glucose production. Annu Rev Nutr.

[30] Wiecek M, Szymura J, Sproull J, Szygula Z. Decreased Blood Asprosin in Hyperglycemic Menopausal Women as a Result of Whole-Body Cryotherapy Regardless of Metabolic Syndrome. J Clin Med. 2019;8(9):1428. 10.3390/jcm8091428. 10.3390/jcm8091428 PMC6780623 31510055

